# Supplementary material for: CD11b+ lung dendritic cells at different stages of maturation induce Th17 or Th2 differentiation
Source: Nat Commun. 2021 Aug 19;12:5029. doi: 10.1038/s41467-021-25307-x (PMC8377117; doi:10.1038/s41467-021-25307-x)
Supplement: Supplementary file 1 — Supplementary information. [file 41467_2021_25307_MOESM1_ESM.pdf]

Supplementary information

CD11b^{+} lung dendritic cells at different stages of maturation induce Th17 or Th2 differentiation

Gentaro Izumi, Hideki Nakano, Keiko Nakano, Gregory S. Whitehead, Sara A. Grimm, Michael B. Fessler, Peer W. Karmaus and Donald N. Cook

Supplementary information contains;

1. Supplementary Figures 1 – 17
2. Supplementary Tables 1 and 2

Supplementary Figure 1

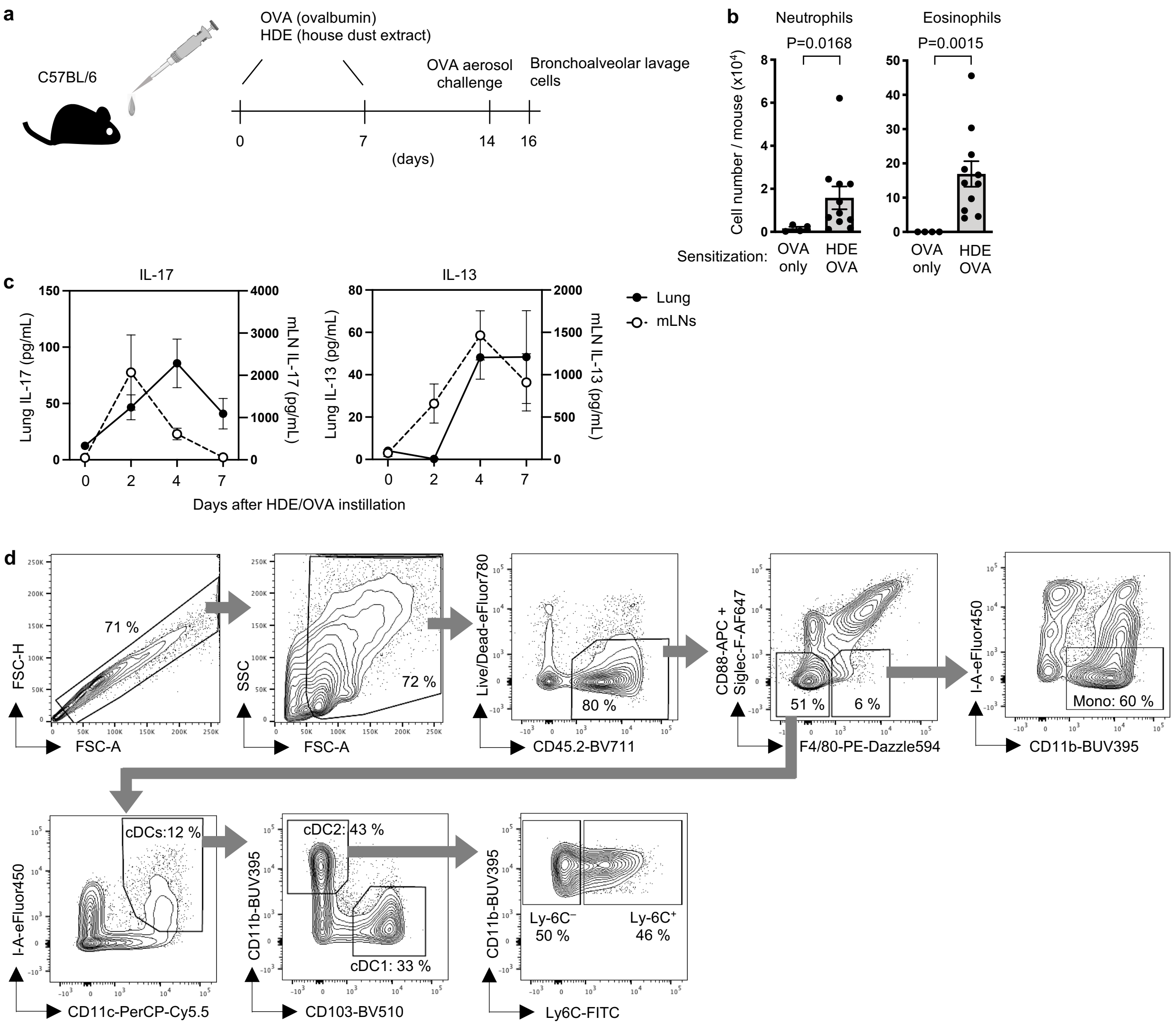

Supplementary Figure 1 Th17 and Th2 responses in the HDE/OVA model of asthma. a Timeline for allergic sensitization and allergen challenge in HDE/OVA-mediated mouse model of asthma. **b** Cell numbers for the indicated leukocyte in BALF of OVA-challenged C57BL/6 mice after sensitization with OVA only or HDE/HDE. Data were analyzed by unpaired two-tailed Mann-Whitney test (*n*=4 in OVA only and *n*=11 in OVA/HDE groups). Data are presented as mean values ± SEM. Source data are provided as a Source Data file. **c** IL-17 and IL-13 production from the lung and mLNs of mice at various times after HDE/OVA inhalation. *n*=6 mice. Data are presented as mean values ± SEM. **d** Gating strategy for flow cytometric analysis of lung cDCs. cDC1: $CD45^+CD11c^+I\text{-}A^+CD11b^-CD103^+CD88^-Siglec\text{-}F^-F4/80^-Live/Dead^-$, cDC2: $CD45^+CD11c^+I\text{-}A^+CD11b^+CD103^-CD88^-Siglec\text{-}F^-F4/80^-Live/Dead^-$. The same gating strategy was used for lung cDC analysis presented on Figure 1f, 4a-d and Supplementary Figure 7e,f.

Supplementary Figure 2

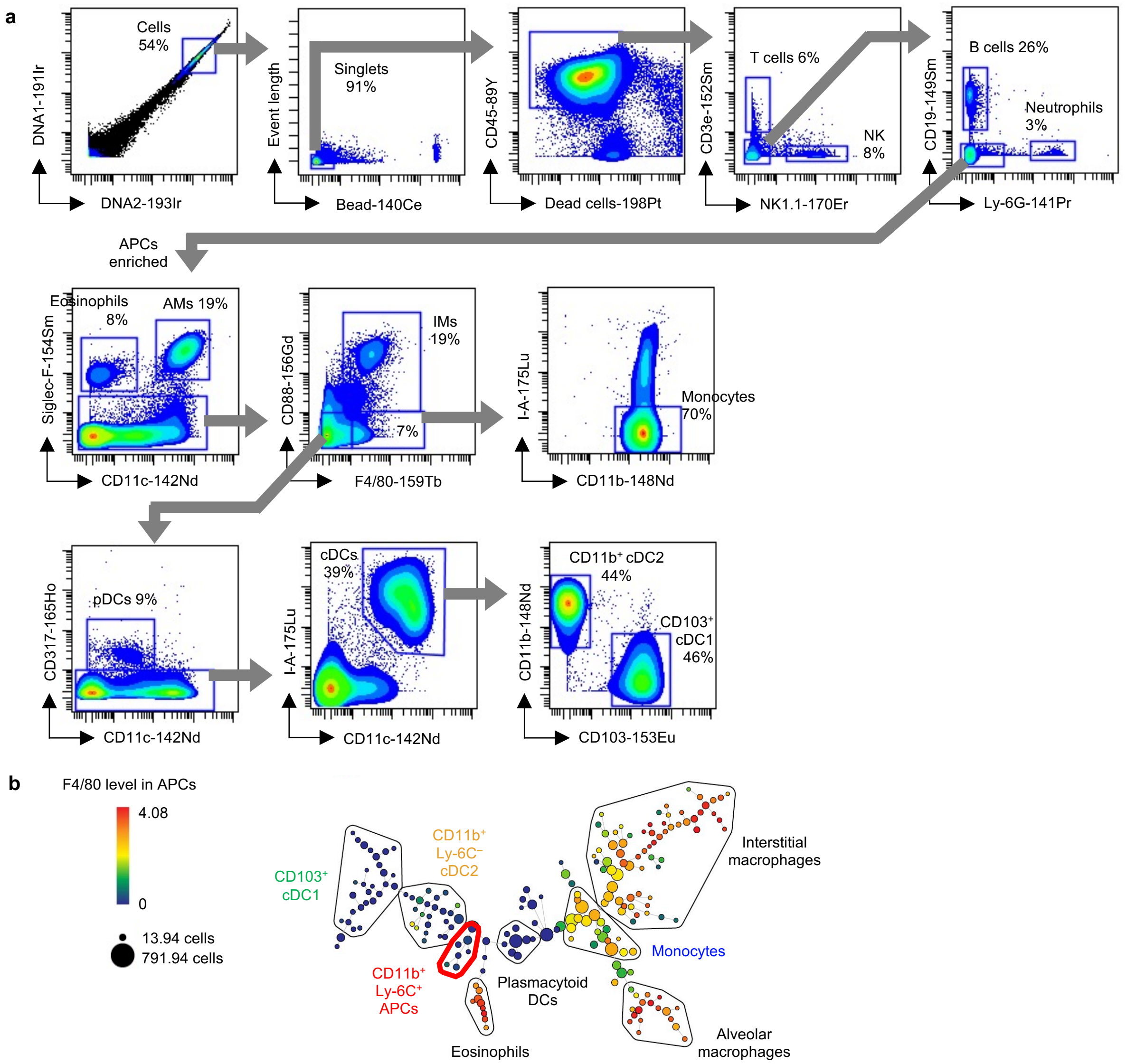

Supplementary Figure 2 Identification of lung Ly-6C⁺ cDC2-like cells. a Gating strategy for mass cytometry analysis of lung leukocytes using the Cytobank platform. cDCs are CD45⁺CD11c⁺**I-A⁺**CD88⁻Siglec-F⁻F4/80⁻CD3e⁻CD19⁻NK1.1⁻Ly-6G⁻. **b** SPADE analysis of lung cells in APCs gates (CD45⁺CD3e⁻CD19⁻NK1.1⁻Ly-6G⁻) including eosinophils after HDE/OVA inhalation. Dot size and color indicate cell number and surface display level of F4/80, respectively.

Supplementary Figure 3

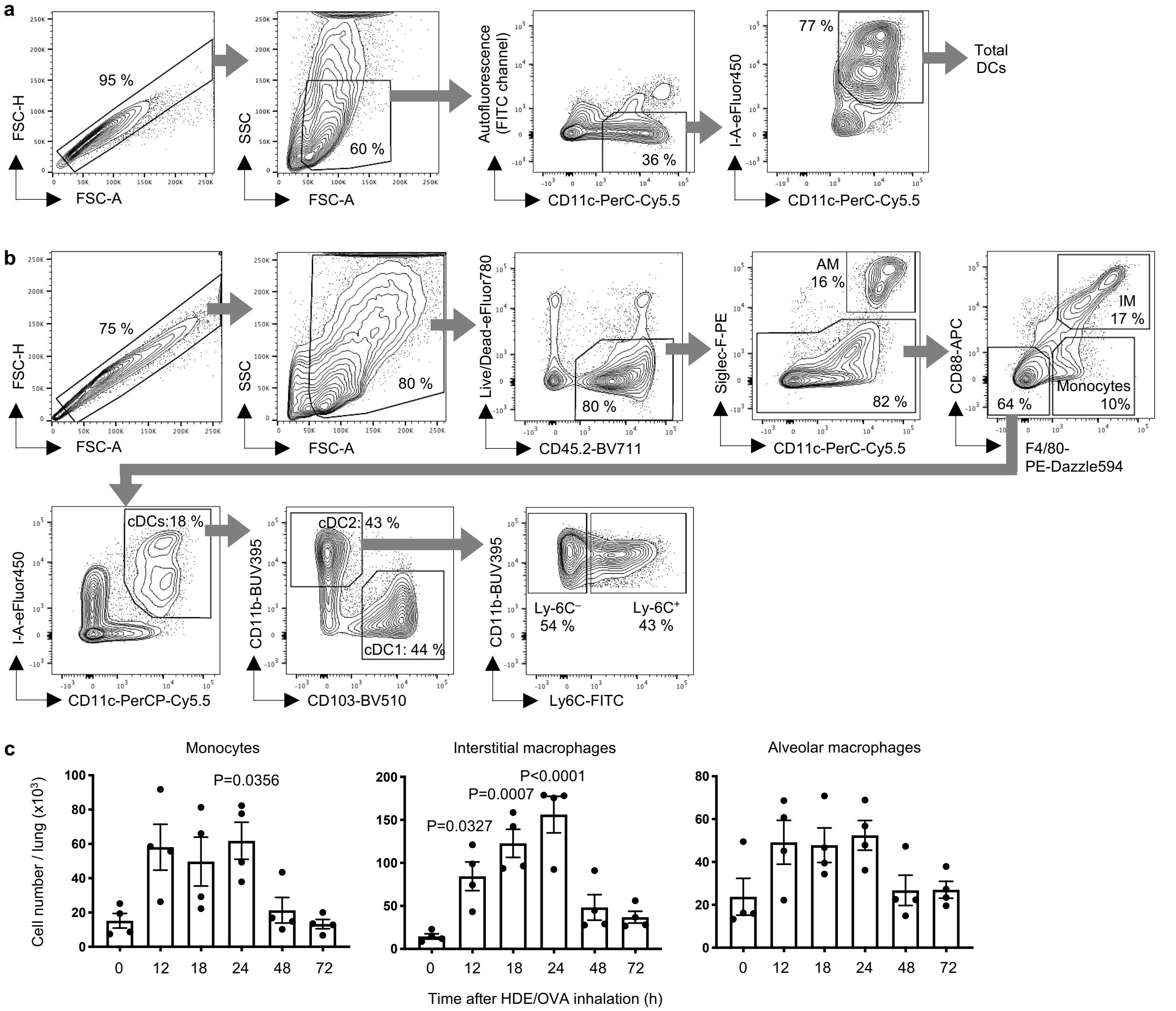

Supplementary Figure 3 Gating strategies for flow cytometry analysis of lung cDCs, monocytes and macrophages upon HDE/OVA inhalation. a Gating strategy for flow cytometric sorting of total lung DCs (CD11c$^+$I-A$^+$ autofluorescence$^-$). The same gating strategy was used for co-culture experiment presented on Fig. 1c. **b** Gating strategy for flow cytometric analysis of lung cDCs, monocytes and macrophages. Monocytes: CD45$^+$CD11b$^+$F4/80$^+$CD88$^-$Siglec-F$^-$Live/Dead$^-$, AMs: CD45$^+$CD11c$^+$Siglec-F$^+$Live/Dead$^-$ and IMs: CD45$^+$CD88$^+$F4/80$^+$Siglec-F$^-$Live/Dead$^-$. The same gating strategy was used for lung DC analysis presented on Figure 1g, 2e,f, Supplementary Figures 3c, 5d and 8a-d. **c** Time course for accumulation and disappearance of monocytes, IMs and AMs in the lung at various times post-HDE/OVA inhalation. Data were analyzed by ordinary one-way ANOVA with Tukey's multiple comparison test (*n*=4). Statistical significance of each time point against steady state is shown. Data are presented as mean values ± SEM. A representative result from two independent experiments is shown. Source data are provided as a Source Data file.

Supplementary Figure 4

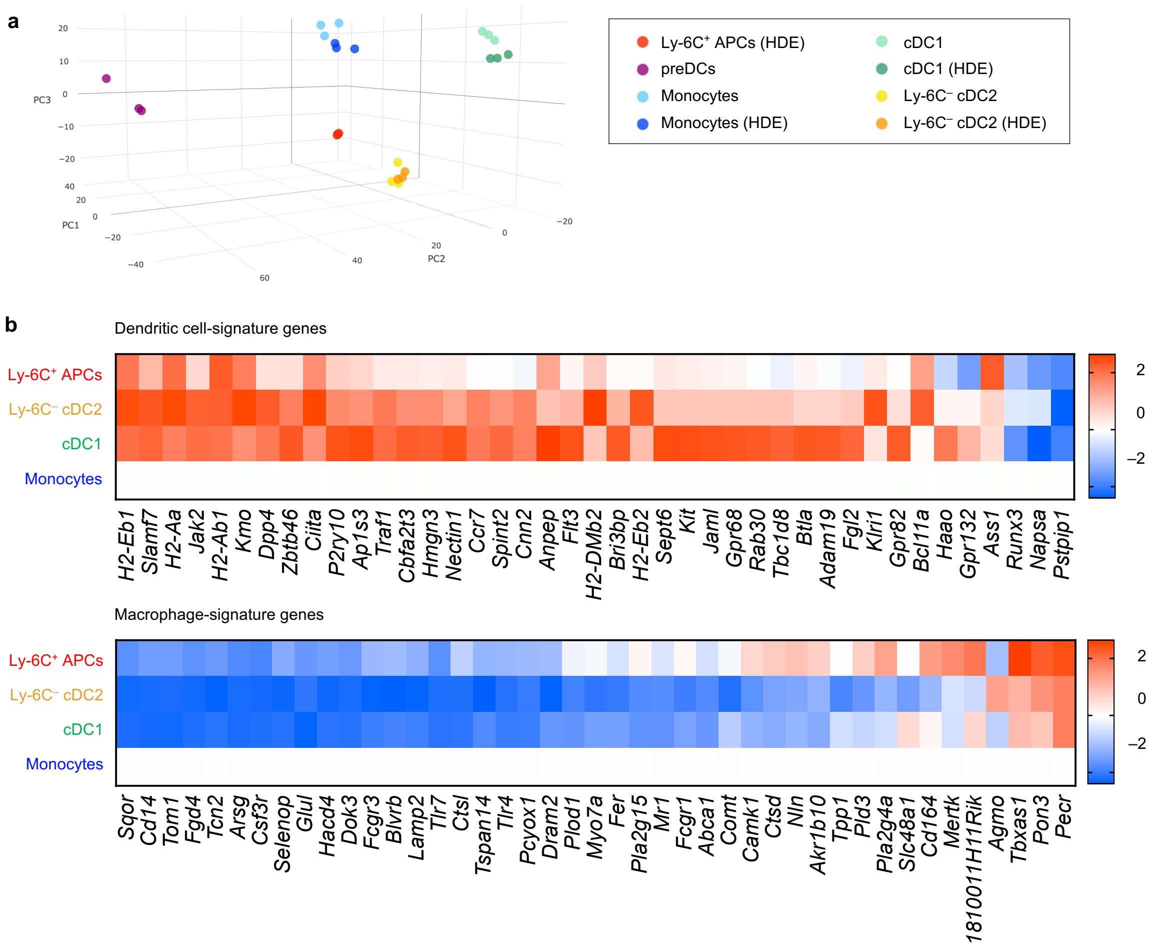

Supplementary Figure 4 Transcriptional profiles of Ly-6C$^+$ APCs. a PCA of RNA-seq data for Ly-6C$^+$CD11b$^+$ (Ly-6C$^+$) APCs and the other indicated APCs from the lungs of untreated or HDE/OVA-treated (HDE) mice. The same data set shown in Fig 2b is displayed from a different angle. **b** Heat map showing expression of DC-signature and macrophage/monocyte-signature genes[35, 36] in the indicated populations after normalization to expression levels in monocytes.

Supplementary Figure 5

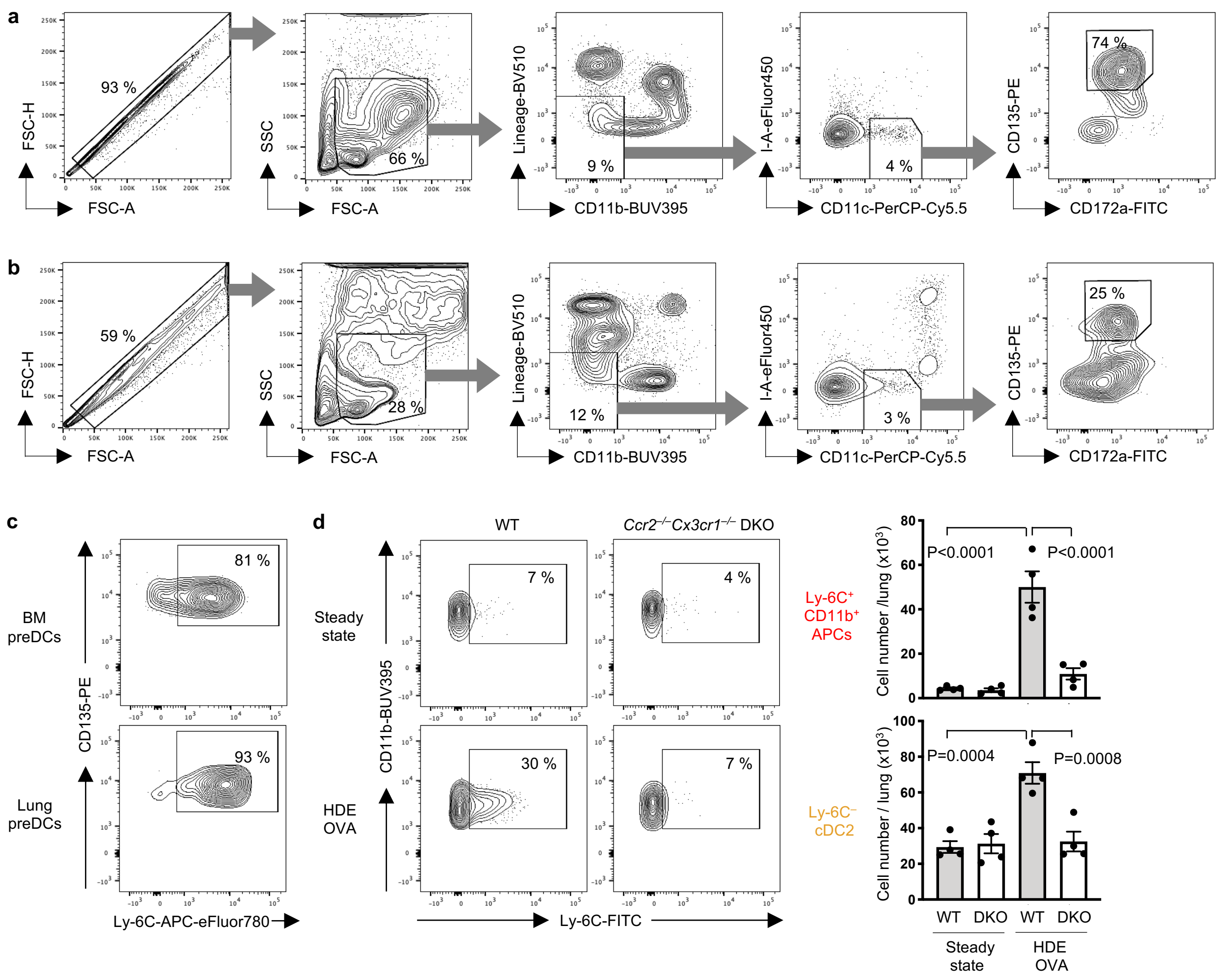

Supplementary Figure 5 Chemokine-dependent accumulation of cDC2 in the lung. a Gating strategy for BM preDC analysis. **b** Gating strategy for lung preDC analysis. Lineage includes CD3ε, CD19, B220, Ly-6G and TER119. The same gating strategy was used for BM preDC sorting in RNA-Seq analysis presented on Figure 2b,d, and BM preDC analysis presented on Supplementary Figure 5c. **c** Flow cytometric analysis of preDCs (Lineage$^-$CD11b$^-$CD11c$^+$CD171a^{int}MHC-II$^-$) from the BM and lung of C57BL/6 mice. A representative result for each tissue is shown from a total of 3 repeats. **d** Flow cytometric analysis of lung cDC2 from WT C57BL/6 and C57BL/6-*Ccr2*$^{-/-}$*Cx3cr1*$^{-/-}$ DKO mice at steady state and following HDE/OVA treatment. The gating strategy is shown in Supplementary Fig. 3b. Data were analyzed by ordinary one-way ANOVA with Sidak's multiple comparison test (n=4). Data are presented as mean values ± SEM. A representative result from two independent experiments is shown. Source data are provided as a Source Data file.

Supplementary Figure 6

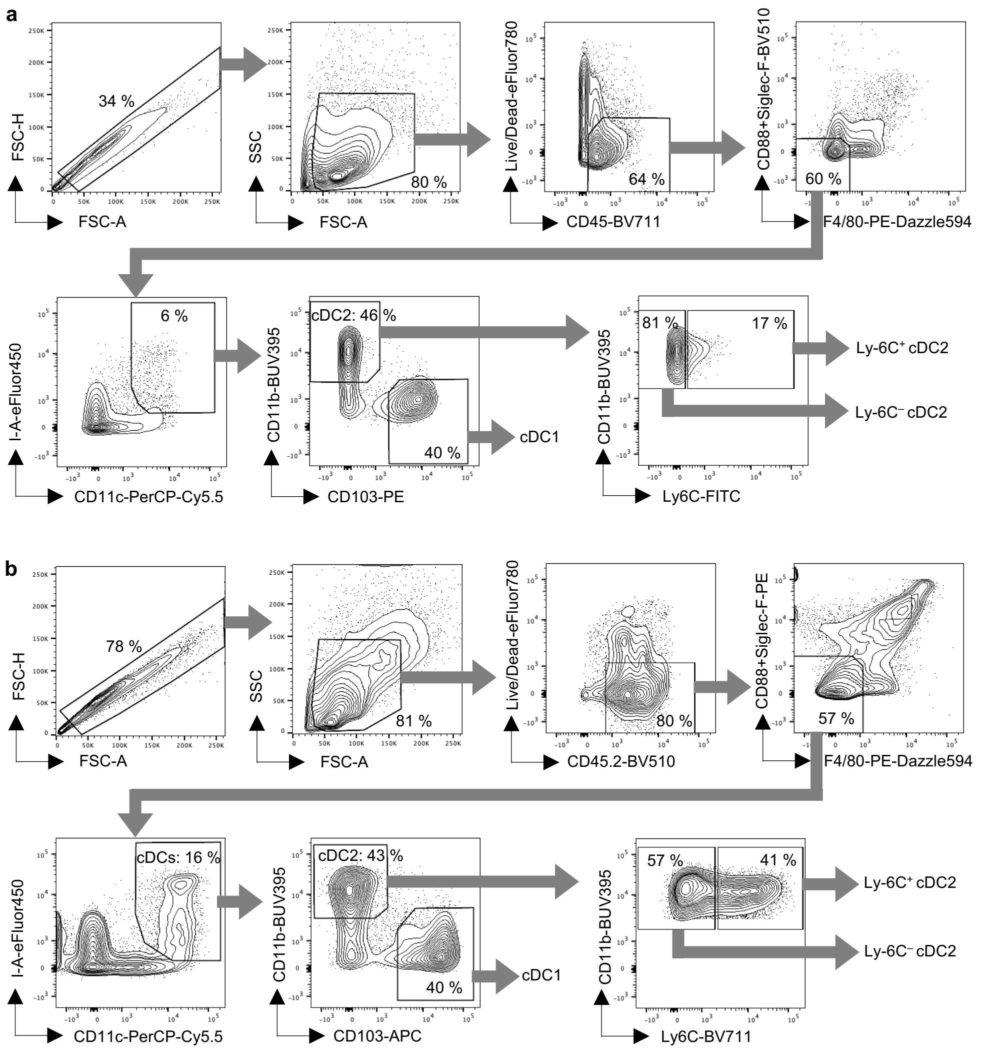

Supplementary Figure 6 Gating strategies for antigen-uptake analyses in flow cytometry. a Gating strategy for the analysis of OVA-AF647 uptake by lung cDC subsets presented on Figure 3a, b. **b** Gating strategy for the analysis of DQ-OVA+ lung cDCs presented on Supplementary Figure 7a, b.

Supplementary Figure 7

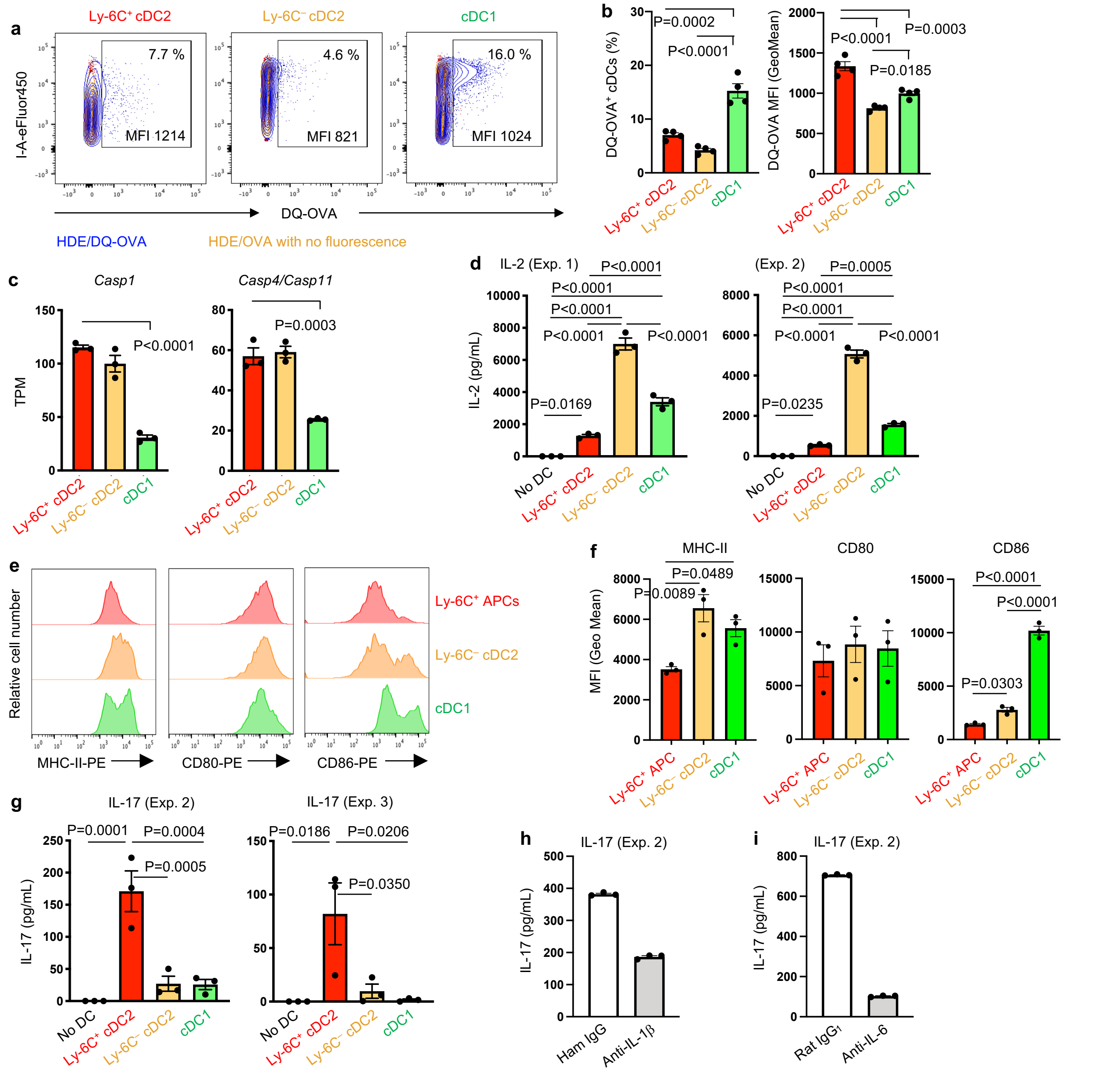

Supplementary Figure 7 APC function of lung cDCs. a, **b** Antigen processing by lung cDCs 16 h after inhalation of DQ-OVA with HDE was analyzed by flow cytometry. The gating strategy is shown in Supplementary Fig. 6b. Representative cytograms (**a**) and compiled data (*n*=4) (**b**) showing cDCs that have processed DQ-OVA. Blue and orange counter plots show cells from mice received DQ-OVA and non-fluorescent OVA, respectively. Data were analyzed by ordinary one-way ANOVA with Tukey's multiple comparison. Data are presented as mean values ± SEM. **c** Expression of *Casp1* and *Casp4/Casp11* in the indicated lung cDC subsets, as determined by bulk RNA-Seq. Data were analyzed by two-way ANOVA with Fisher's LSD multiple comparison test (*n*=3). Data are presented as mean values ± SEM. **d** IL-2 production from OT-II CD4$^+$ T cells co-cultured with purified lung cDCs. IL-2 in the culture supernatant on day 3 was measured by ELISA. **e**, **f** Representative histograms (**e**) and compiled data (**f**) showing surface display levels of MHC-II (I-A/I-E), CD80 and CD86 on lung cDC subsets. Gating strategies are shown in Supplementary Fig. 1d. Data were analyzed by ordinary one-way ANOVA with Tukey's multiple comparison test (*n*=3). Data are presented as mean values ± SEM. **g** IL-17, measured by ELISA, in supernatants of CD4$^+$ T cells cultured with lung cDCs. Data were analyzed by ordinary one-way ANOVA with Tukey's multiple comparison test (*n*=3). Data are presented as mean values ± SEM. **h**,**i** IL-17 production from CD4$^+$ T cells stimulated by Ly-6C$^+$ cDC2 in the presence of anti-IL-1b (**h**) or -IL-6 (**i**) neutralizing Abs or isotype control. IL-17 in the supernatant was measured by ELISA in triplicate. Data are presented as mean values ± SEM. Source data are provided as a Source Data file.

Supplementary Figure 8

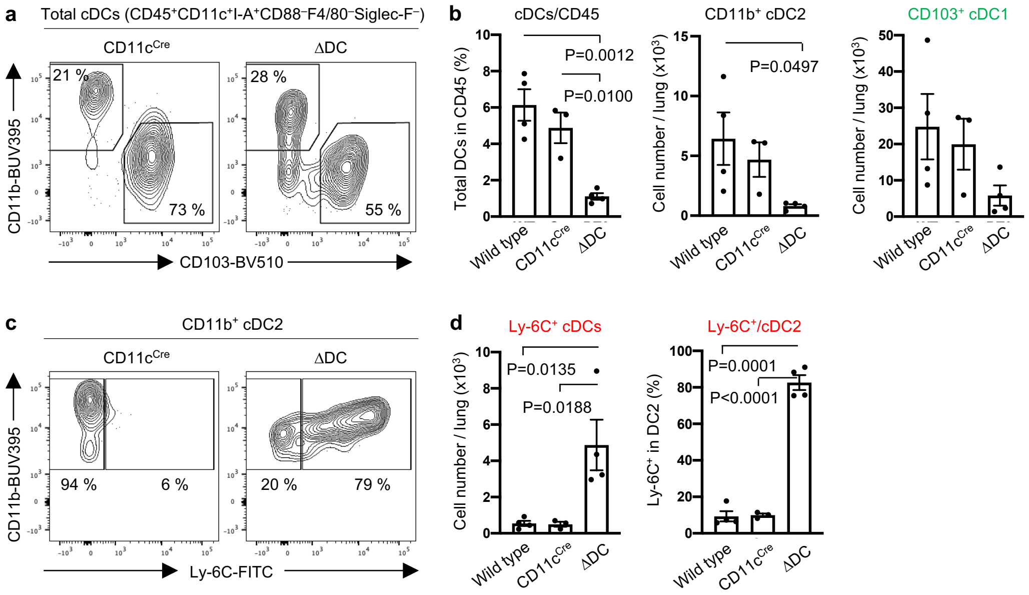

Supplementary Figure 8 Ly-6C⁺ cDCs in ΔDC mice. a-d Analysis of cDCs (CD45⁺CD11c⁺I-A⁺CD88⁻F4/80⁻Siglec-F⁻Live/Dead⁻) in lungs of WT, CD11c^{Cre} and ΔDC mice (C57BL/6-background)) at steady state. The gating strategy is shown in Supplementary Fig. 3b. Representative cytograms (**a**, **c**) and compiled data (**b**, **d**) for percentages and cell numbers for total cDCs, cDC2, and cDC1 (**b**) and for Ly-6C⁺ cDC2 (**d**) are shown. Data were analyzed by ordinary one-way ANOVA with Dunnett's multiple comparison test (*n*=4 wild type and ΔDC mice, and *n*=3 CD11c^{Cre} mice). Data are presented as mean values ± SEM. Source data are provided as a Source Data file.

Supplementary Figure 9

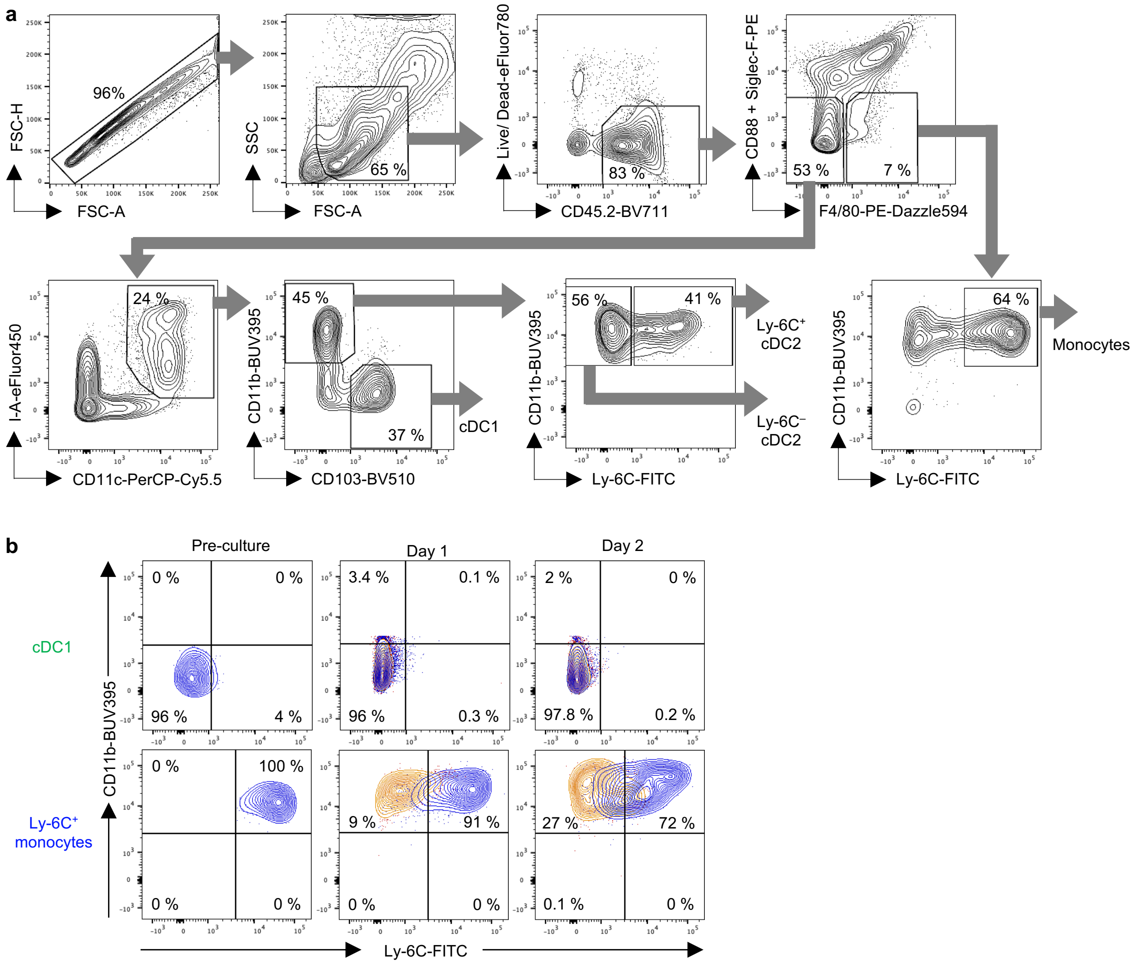

Supplementary Figure 9 Surface Ly-6C on cDC subsets and monocytes. a Gating strategy for sorting of cDCs and monocytes and flow cytometric analysis of cultured cells presented on Figure 5a,b. **b** Representative cytograms of freshly isolated cDC1 and monocytes, and after *ex vivo* culture are shown. Blue and orange counter plots show the results stained with specific Ab and isotype control rat IgM, respectively.

Supplementary Figure 10

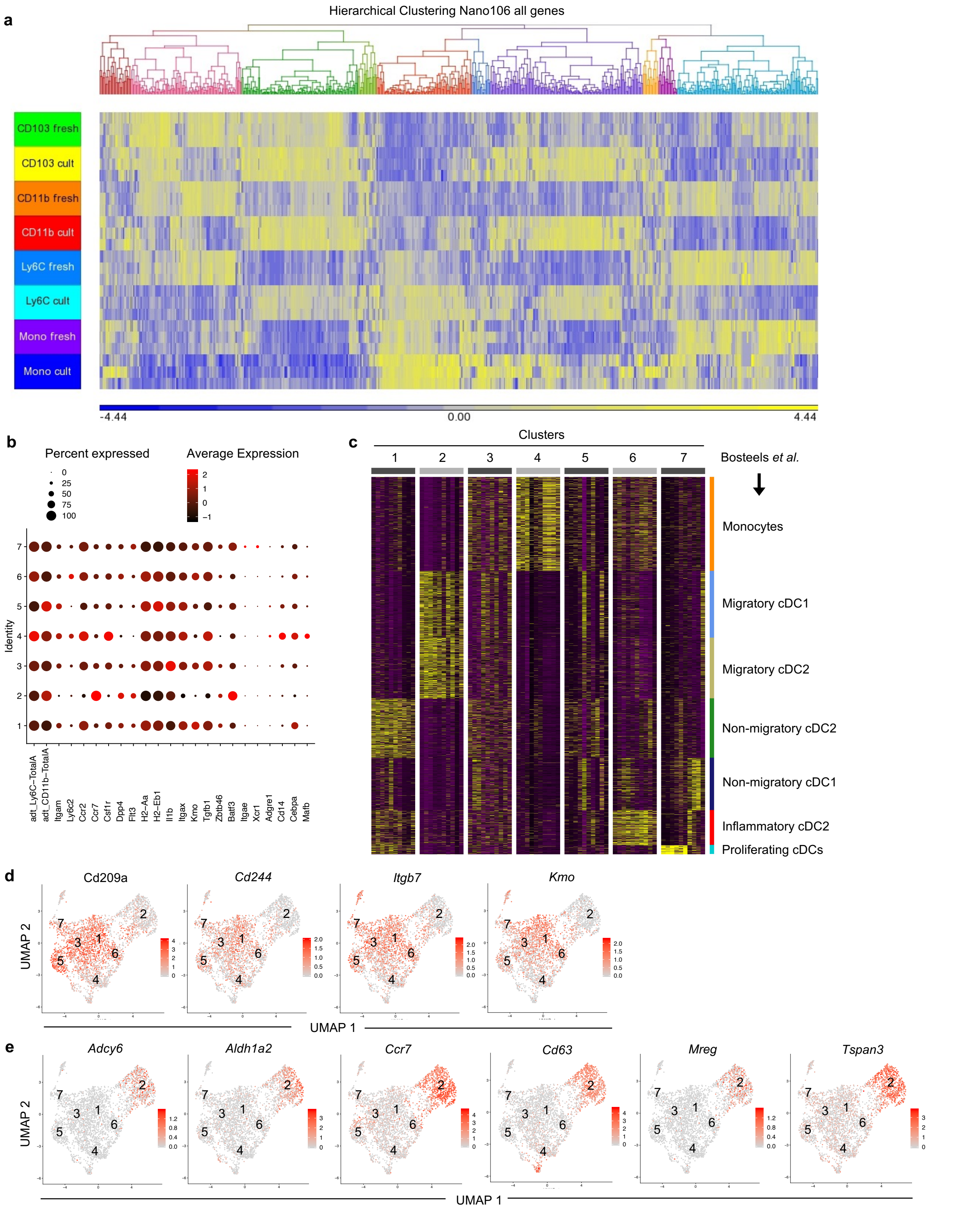

Supplementary Figure 10 Transcriptomic analysis of lung cDC2 subpopulations. a Heatmap of gene expression, as measured by Nanostring nCounter Immunology panel, for freshly isolated Ly-6C^{+}CD11b^{+} cDC2 (Ly6C), Ly-6C^{-}CD11b^{+} cDC2 (CD11b), CD103^{+} cDC1 and monocytes (mono), and for cells after 2 days culture. **b** Expression of cDC- and macrophage-signature genes in the 7 CD11b^{+} cell clusters, based on Seurat analysis of scRNA-Seq data. **c** Heatmap comparing expression of 10 diagnostic DEGs for each of the seven clusters shown in Fig. 5d, with clusters identified by Bosteels *et al.*[25]. **d** cDC-signature genes expressed in four cDC2 clusters; 1, 3, 5 and 6 analyzed in scRNA-Seq. **e** Representative genes exclusively expressed in cluster 2.

Supplementary Figure 11

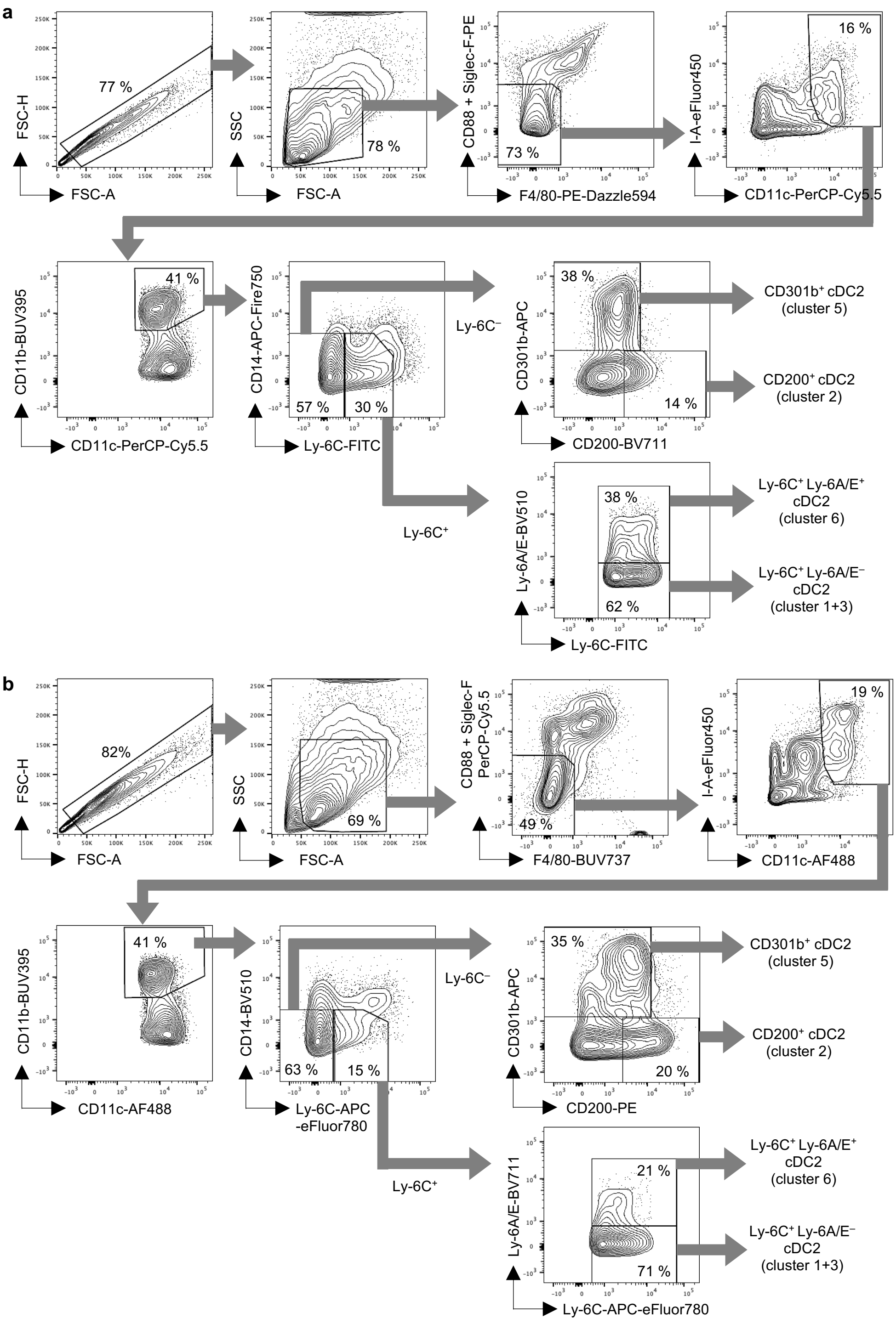

Supplementary Figure 11 Gating strategies for flow cytometry analyses of lung cDC2 subpopulations. **a** Gating strategy for lung cDC2 subpopulation analysis presented on Fig. 6c. **b** Gating strategy for lung cDC2 subpopulation analysis presented on Fig. 6d and Supplementary Fig. 12a, and for lung DC sorting in an analysis presented on Figure 8b,c.

Supplementary Figure 12

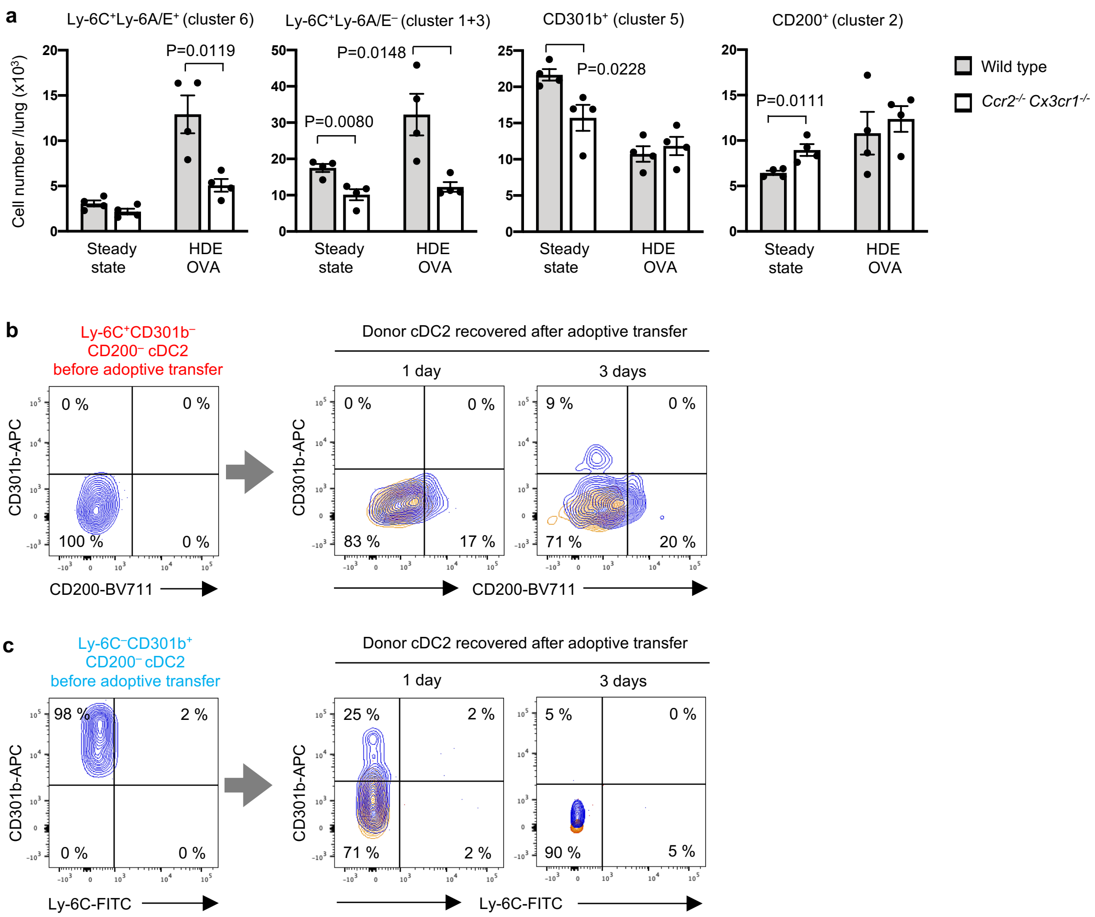

Supplementary Figure 12 Chemokine-dependent accumulation and maturation of lung cDC2 subpopulations. a Flow cytometric analysis of cDC2 subpopulations in lungs of WT C57BL/6 and C57BL/6-*Ccr2*$^{-/-}$*Cx3cr1*$^{-/-}$ DKO mice at steady state and at 16 h post-HDE/OVA inhalation. The gating strategy is shown in Supplementary Fig. 11b. Data were analyzed by unpaired two-tailed t-test (*n*=4). Data are presented as mean values ± SEM. Source data are provided as a Source Data file. **b**, **c** Flow cytometric analysis of Ly-6C^{+}CD301b^{-}CD200^{-} (**b**) or Ly-6C^{-}CD301b^{+}CD200^{-} (**c**) before and 1 day and 3 days after adoptive transfer. The gating strategies for cell sorting and analysis in flow cytometry are shown in Supplementary Fig. 13a,b. Blue and orange counter plots show cells stained with specific Abs and isotype controls, respectively. A representative result from two independent experiments is shown.

Supplementary Figure 13

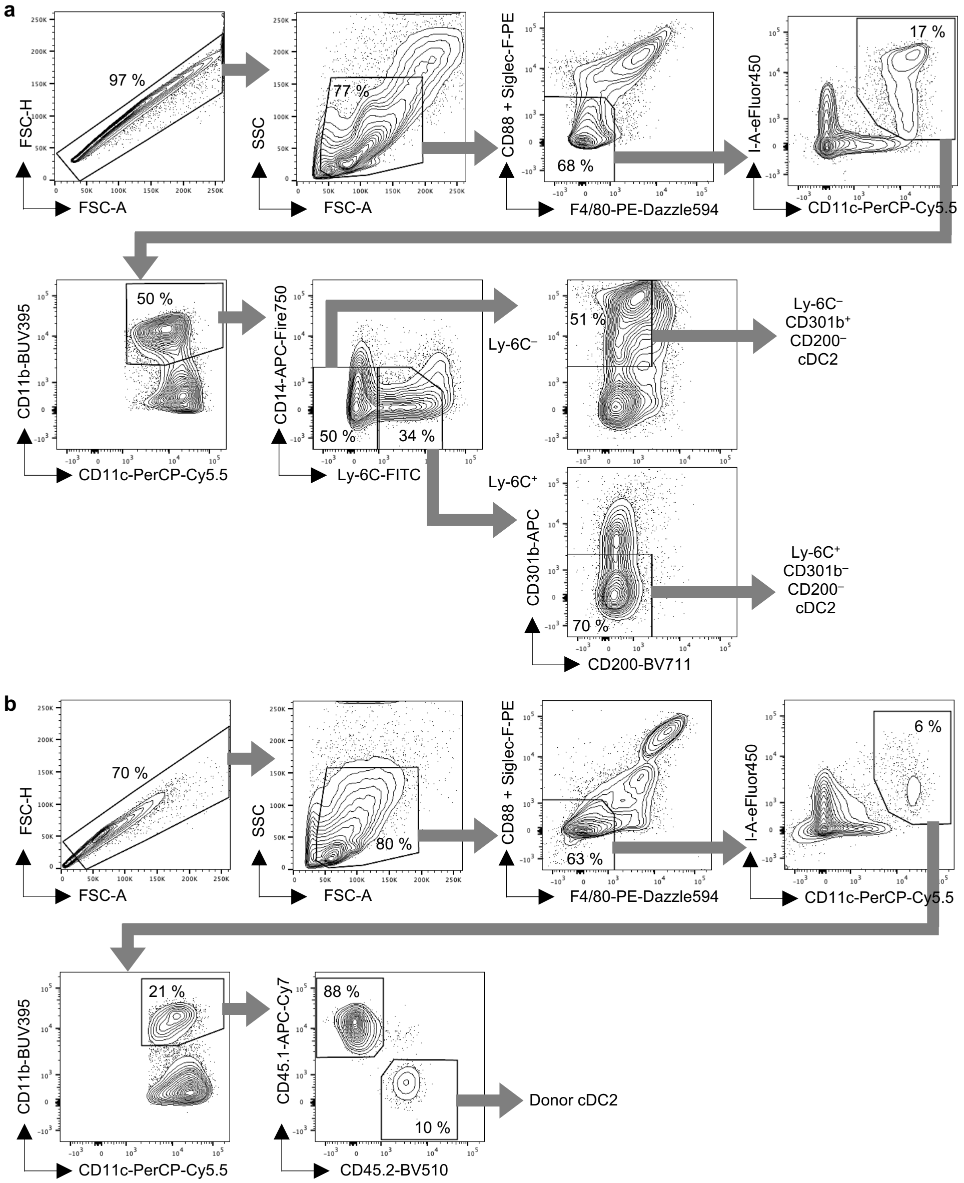

Supplementary Figure 13 Gating strategies for flow cytometry analyses of lung cDC2. a Gating strategy for sorting of Ly-6C$^+$ and CD301b$^+$ cDC2 for adoptive transfer presented on Figure 7d,e and Supplementary Figure 12b,c. **b** Gating strategy for the analysis of adoptive transferred cDC2 (CD45.2$^+$) in the recipient lung presented on Figure 7d,e and Supplementary Figure 12b,c.

Supplementary Figure 14

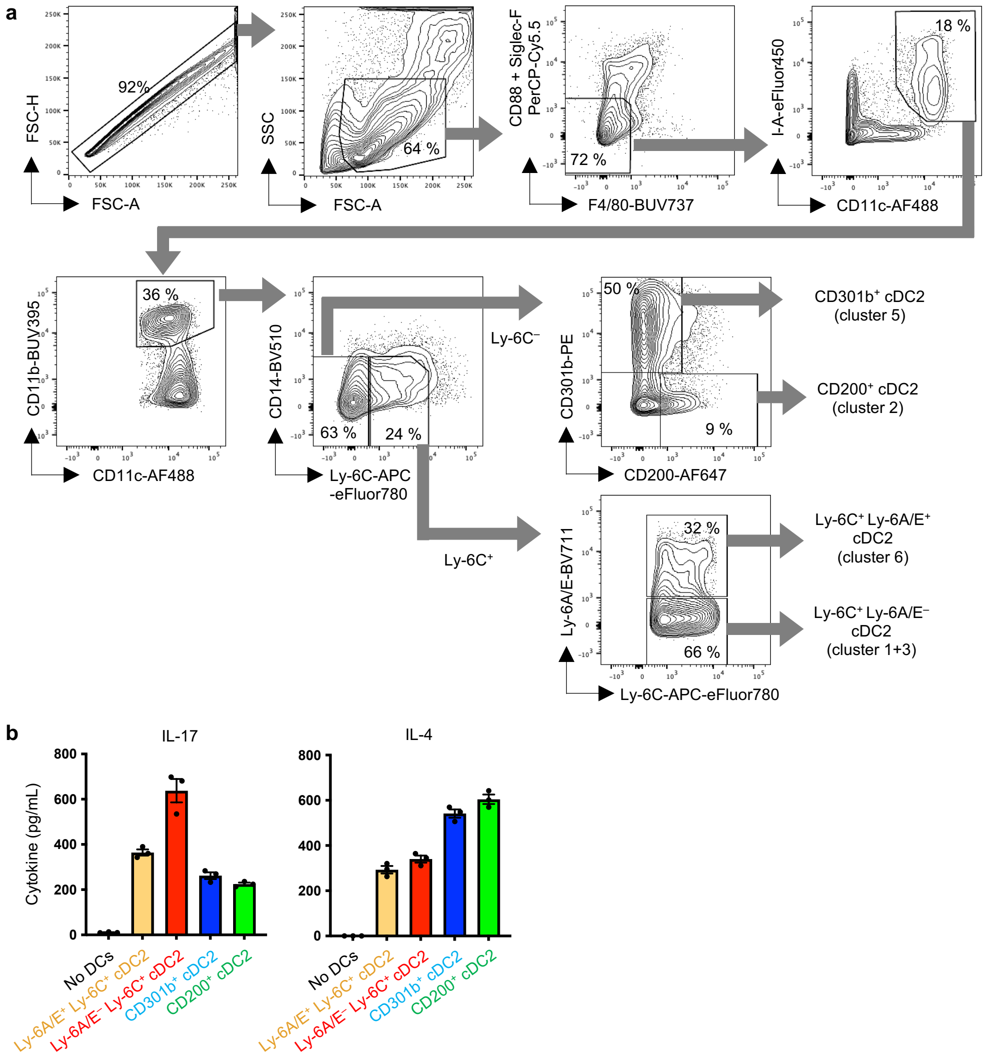

Supplementary Figure 14 Select CD11b⁺ cDC2 subpopulations stimulate Th17 differentiation. a Gating strategy for lung DC2 sorting in the analysis presented on Figure 8b,c, and Supplementary Figure 14b. **b** IL-17 and IL-4 production of OT-II CD4⁺ T cells stimulated by indicated cDC2 subpopulations. The gating strategy for cDC sorting is shown in Supplementary Fig. 14a. Cytokines in the supernatant were measured by ELISA in triplicate. Data are presented as mean values ± SEM. Source data are provided as a Source Data file.

Supplementary Figure 15

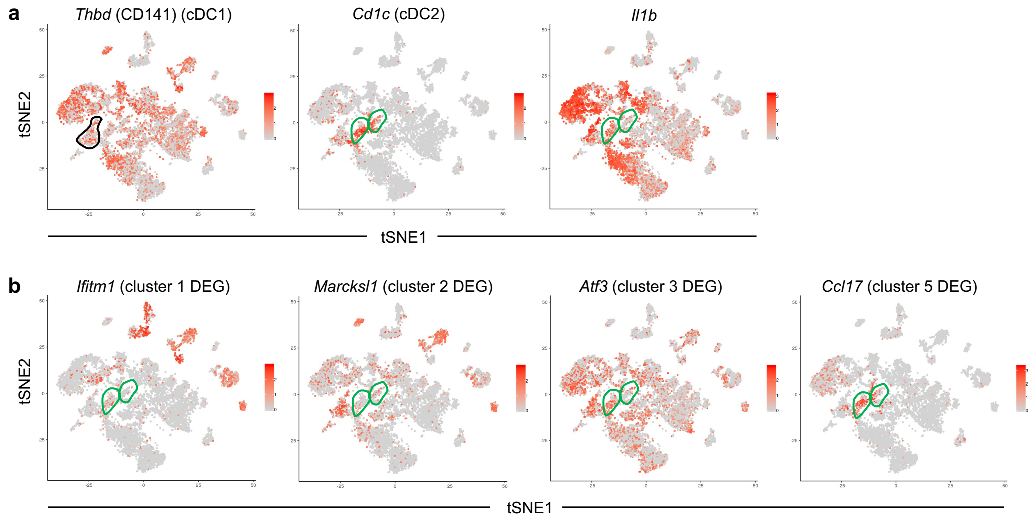

Supplementary Figure 15 Gene expression in human cDC2 subpopulations. a, **b** Select gene expression in human lung cDCs. Non-epithelial cell clusters for human lungs were obtained from the human atlas reported by Vieira Braga *et al*[50]. Black and green lines indicate cDC1 and cDC2, respectively. (**a**) tSNE plots showing the expression of cDC1- (*Tnbd*) and cDC2- (*Cd1c*) signature genes, and *Il1b*. (**b**) tSNE plots showing the expression of mouse cDC2 cluster DEGs in human lung cells. Expression levels are indicated by color difference.

Supplementary Figure 16

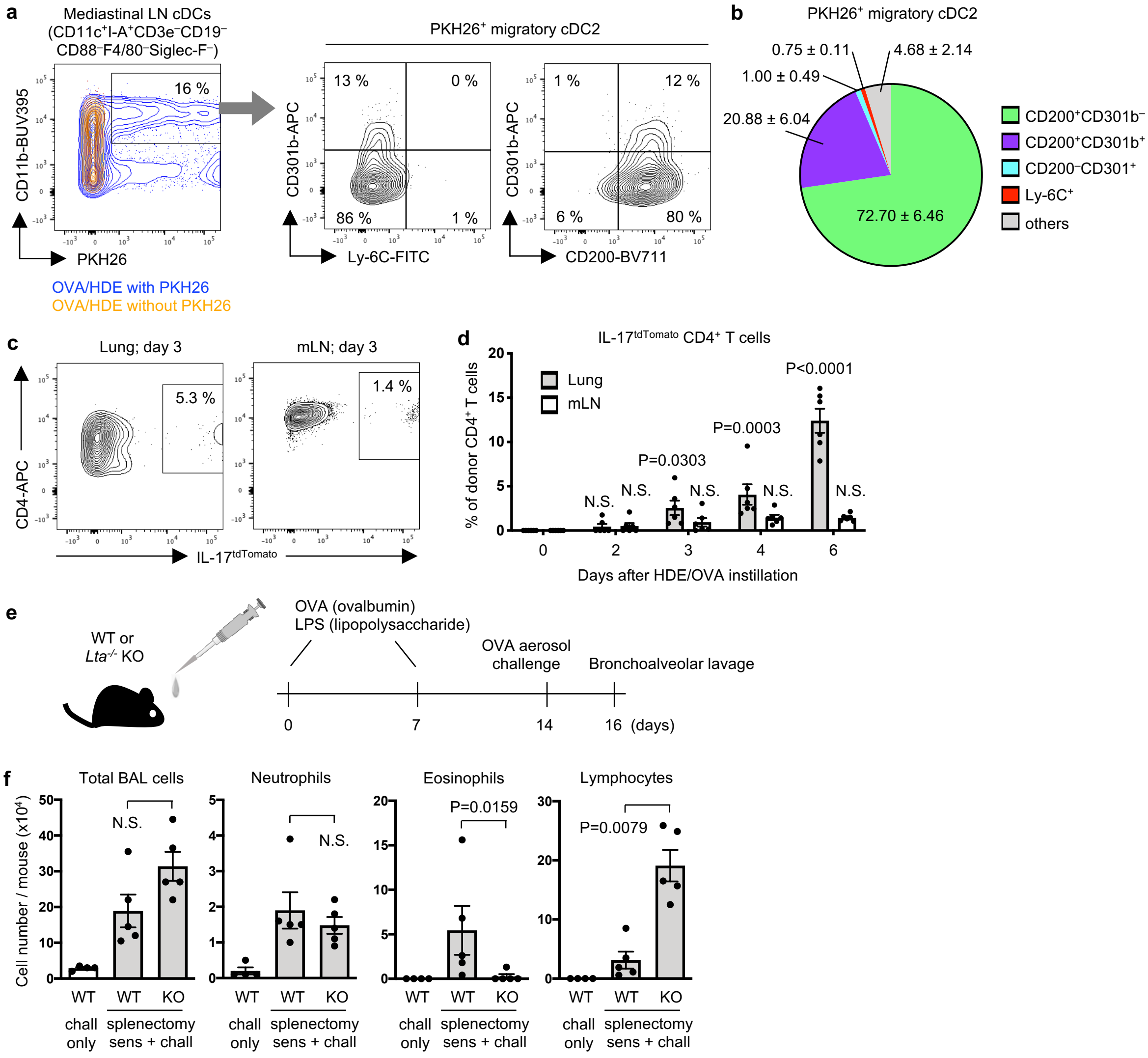

Supplementary Figure 16 Th17 responses are induced in the lung. a, **b** Migratory cDC2 in mLNs. cDC2 subsets in mLNs of mice sensitized with HDE/OVA together with PKH26 (blue) or without PKH26 (orange) were analyzed by flow cytometry 24 h post-instillation. The gating strategy is shown in Supplementary Fig. 17a. Representative cytograms (**a**), and compiled data (**b**) of PKH26⁺ migratory cDC2 are shown. Data are presented as mean values ± SD (*n*=4). **c**, **d** Th17 cells (*Il17*-tdTomato⁺) in the lung and mLNs of C57BL/6-CD45.1 recipient mice after adoptive transfer of *Il17* fate-mapping OT-II T cells (CD45.2⁺) followed by HDE/OVA inhalation. Representative cytograms (**c**), and compiled data (**d**) showing frequencies of tdTomato⁺ cells among CD45.2⁺ donor (C57BL/6) CD4⁺ T cells before at indicated days after HDE/OVA instillation. Gating strategies for cell sorting and analysis in flow cytometry are shown in Supplementary Fig. 17b,c. Data were analyzed by two-way ANOVA with Dunnett's multiple comparison test (*n*=6). Statistical significance of each time point against steady state is shown. Data are presented as mean values ± SEM. N.S.: not significant. **e** Timeline of LPS/OVA-mediated mouse model of asthma employing WT C57BL/6 or C57BL/6-LTa KO mice. **f** Cell numbers of BALF leukocytes following challenge. Mice undergoing splenectomy prior to sensitization are indicated. Data were analyzed by two-tailed Mann-Whitney test (*n*=4 WT chall only, and *n*=5 sens + chall groups). Data are presented as mean values ± SEM. Statistical significance denotes comparisons between WT and KO mice. N.S.: not significant. Source data are provided as a Source Data file.

Supplementary Figure 17

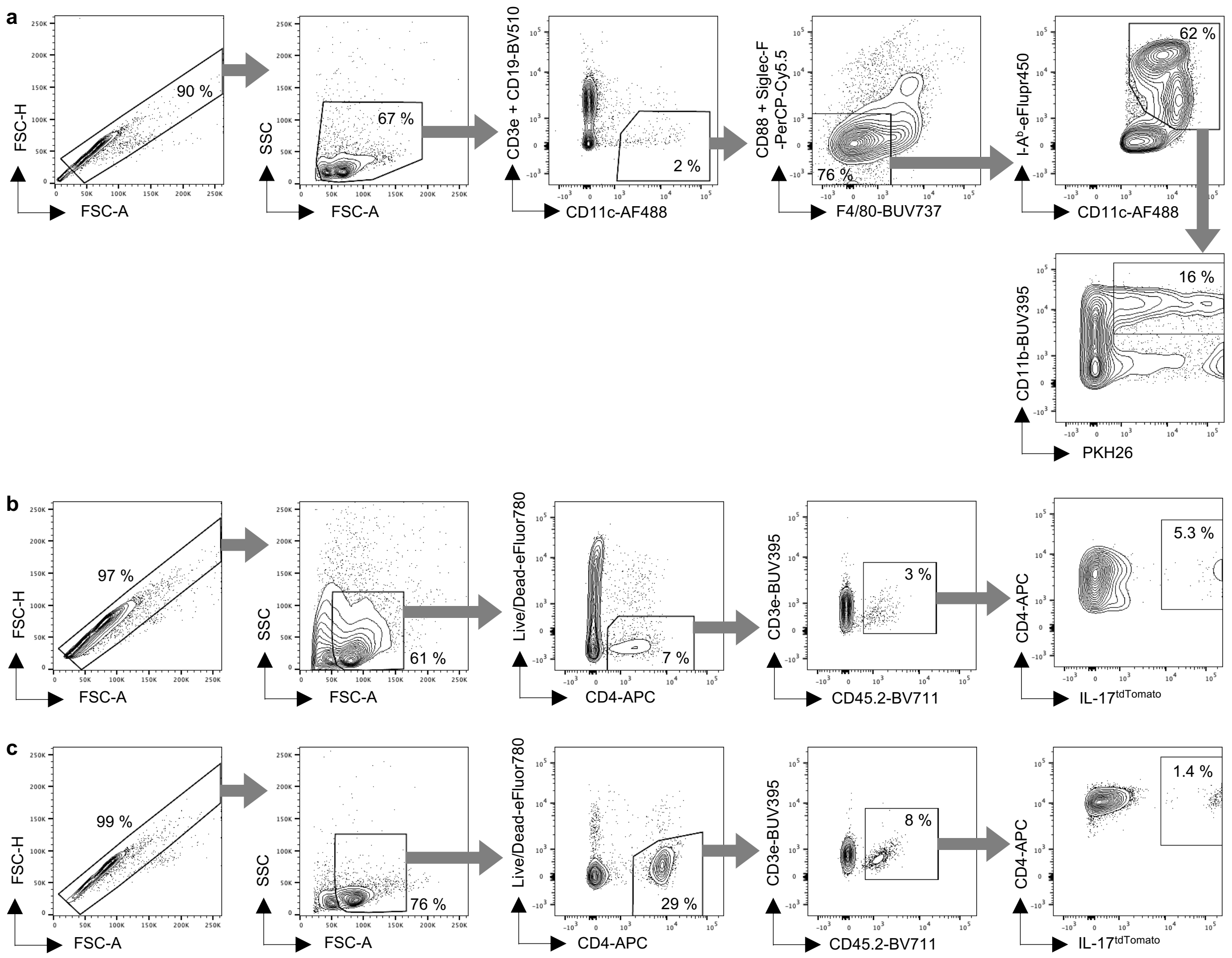

Supplementary Figure 17 Gating strategies for flow cytometry analyses of migratory cDCs and IL-17 reporter T cells. a Gating strategy for the analysis of PKH26⁺ migratory cDCs in the mLNs after inhalation of HDE/OVA together with PKH26 dye. This gating strategy was used an analysis presented on Supplementary Figure 16a,b. **b**, **c** Gating strategy for the analysis of CD4⁺ T cells derived from IL-17-fate-mapping OT-II TCR transgenic donor mice (CD45.2) in the lung (**b**) or mLNs (**c**). These gating strategies were used an analysis presented on Supplementary Figure 16c,d.

Supplementary Table 1. Antibodies used for mass cytometry

Target molecules	Clone	Label	Source	Catalog number	Dilution
CD45	30-F11	89Y	Fluidigm	3089005B	1:200
Ly-6G	1A8	141Pr	Fluidigm	3141008B	1:200
CD11c	N418	142Nd	Fluidigm	3142003B	1:100
CD43	S11	146Nd	Fluidigm	3146009B	1:200
CD11b	M1/70	148Nd	Fluidigm	3148003B	1:100
CD19	6D5	149Sm	Fluidigm	3149002B	1:200
CD115	AFS98	150Nd	eBioscience & Fluidigm	custom order	1:100
Ly-6C	HK1.4	151Eu	Lederer Lab, Harvard	custom order	1:400
CD3e	145-2C11	152Sm	Fluidigm	3152004B	1:200
CD103	2.00E+07	153Eu	Biolegend, Fluidigm	custom order	1:400
Siglec-F	E50-2440	154Sm	BD Bioscience & Fluidigm	custom order	1:400
CD326/EpCAM	G8.8	155Gd	Fluidigm	custom order	1:100
CD88	20/70	156Gd	Biolegend & Fluidigm	custom order	1:100
F4/80	BM8	159Tb	Fluidigm	3159009B	1:100
CD40	HM40-3	161Dy	Fluidigm	3161020B	1:200
CD24	M1/69	162Dy	Lederer Lab, Harvard	custom order	1:100
CX3CR1	SA011F11	164Dy	Fluidigm	3164023B	1:400
CD317/BST2	927	165Ho	BD Bioscience & Fluidigm	custom order	1:100
CD86	GL-1	166Er	Lederer Lab, Harvard	custom order	1:100
CD64	X54-5/7.1	168Er	Lederer Lab, Harvard	custom order	1:100
CD172a	P84	169Tm	Lederer Lab, Harvard	custom order	1:200
CD161/NK1.1	PK136	170Er	Fluidigm	3170002B	1:100
CD80	16-10A1	171Yb	Fluidigm	3171008B	1:200
MHC-II I-Ab	AF6-120.1	175Lu	eBioscience	custom order	1:100
XCR1	ZET	APC	BioLegend	148205	1:200
MMR	MR5D3	biotin	BioLegend	123003	1:100
CD26	H194-112	FITC	BioLegend	137805	1:200
CD83	Michel-19	PE	BD Bioscience	558205	1:100
APC	APC003	176Yb	Fluidigm	3176007B	1:100
Biotin	1D4-C5	143Nd	Fluidigm	3143008B	1:100
FITC	FIT-22	144Nd	Fluidigm	3144006B	1:100
PE	PE001	145Nd	Fluidigm	3145006B	1:100

Fluorochrome- or biotin-conjugated Abs were followed by metal-conjugated secondary Abs.

Supplementary Table 2. Cluster DEGs in moue lung cDC2 scRNA-Seq analysis

ID	Gene	Cluster	ID	Gene	Cluster
1	Mdh2	1	41	Mgl2	5
2	Napsa	1	42	Ear2	5
3	Ccnd3	1	43	Clec4b1	5
4	Klrd1	1	44	Trf	5
5	Ckb	1	45	Cst3	5
6	Cfp	1	46	Mt1	5
7	Cd300a	1	47	Wfdc17	5
8	Cd209a	1	48	Cd209a	5
9	Cd7	1	49	Ccl17	5
10	Ifitm1	1	50	Retnla	5
11	Fscn1	2	51	Ifit3	6
12	Ccr7	2	52	Ifi204	6
13	Tmem123	2	53	Mnda	6
14	Socs2	2	54	Ifi205	6
15	Tspan3	2	55	Irf7	6
16	Fabp5	2	56	Isg15	6
17	Ccl22	2	57	Ms4a4c	6
18	Marcksl1	2	58	Ly6a	6
19	Apol7c	2	59	Plac8	6
20	Ccl5	2	60	Cxcl10	6
21	Il1b	3	61	2810417H13Rik	7
22	Nr4a1	3	62	Top2a	7
23	Btg2	3	63	Stmn1	7
24	Cd83	3	64	Dut	7
25	Pim1	3	65	Dctpp1	7
26	Ifrd1	3	66	Hmgb2	7
27	Pmaip1	3	67	Ifi205	7
28	Socs3	3	68	Naaa	7
29	Atf3	3	69	Ppt1	7
30	Tnip3	3	70	Irf8	7
31	Lyz2	4			
32	Cebpb	4			
33	Tgfbi	4			
34	Fn1	4			
35	Clec4n	4			
36	Ccl6	4			
37	C1qc	4			
38	C1qb	4			
39	C1qa	4			
40	Apoe	4			
