# Supplementary material for: CD11b+ lung dendritic cells at different stages of maturation induce Th17 or Th2 differentiation
Source: Nat Commun. 2021 Aug 19;12:5029. doi: 10.1038/s41467-021-25307-x (PMC8377117; doi:10.1038/s41467-021-25307-x)
Supplement: Supplementary file 3 — Description of Additional Supplementary Files. [file 41467_2021_25307_MOESM3_ESM.pdf]

Description of Additional Supplementary Files

File Name: Supplementary Data 1
Description: mRNA expression was examined using the NanoString platform utilizing the Mouse Myeloid Innate Immunity Panel v2 (Nanostring Technologies). RNA expression was quantified on the nCounter Digital Analyzer. Data were adjusted utilizing the manufacturer's positive and negative experimental control probes as well as housekeeping genes with nSolver 4.0 software (Nanostring Technologies). The results (log2) are presented.

File Name: Supplementary Data 2
Description: The differentially expressed gene (DEG) tests were performed with DESeq2. The test was two-sided, and the p-values were adjusted by the Benjamini-Hochberg method.
