# Supplementary material for: CD11b+ lung dendritic cells at different stages of maturation induce Th17 or Th2 differentiation
Source: Nat Commun. 2021 Aug 19;12:5029. doi: 10.1038/s41467-021-25307-x (PMC8377117; doi:10.1038/s41467-021-25307-x)
Supplement: Supplementary file 6 — Reporting summary. [file 41467_2021_25307_MOESM6_ESM.pdf]

nature research

Corresponding author(s): Hideki Nakano

Last updated by author(s): Apr 26, 2021

Reporting Summary

Nature Research wishes to improve the reproducibility of the work that we publish. This form provides structure for consistency and transparency in reporting. For further information on Nature Research policies, see Authors & Referees and the Editorial Policy Checklist .

Statistics

For all statistical analyses, confirm that the following items are present in the figure legend, table legend, main text, or Methods section.

n/a	Confirmed	
☐	☒	The exact sample size (*n*) for each experimental group/condition, given as a discrete number and unit of measurement
☐	☒	A statement on whether measurements were taken from distinct samples or whether the same sample was measured repeatedly
☐	☒	The statistical test(s) used AND whether they are one- or two-sided<br>*Only common tests should be described solely by name; describe more complex techniques in the Methods section.*
☐	☒	A description of all covariates tested
☐	☒	A description of any assumptions or corrections, such as tests of normality and adjustment for multiple comparisons
☐	☒	A full description of the statistical parameters including central tendency (e.g. means) or other basic estimates (e.g. regression coefficient) AND variation (e.g. standard deviation) or associated estimates of uncertainty (e.g. confidence intervals)
☐	☒	For null hypothesis testing, the test statistic (e.g. *F*, *t*, *r*) with confidence intervals, effect sizes, degrees of freedom and *P* value noted<br>*Give P values as exact values whenever suitable.*
☒	☐	For Bayesian analysis, information on the choice of priors and Markov chain Monte Carlo settings
☒	☐	For hierarchical and complex designs, identification of the appropriate level for tests and full reporting of outcomes
☒	☐	Estimates of effect sizes (e.g. Cohen's *d*, Pearson's *r*), indicating how they were calculated

Our web collection on statistics for biologists contains articles on many of the points above.

Software and code

Policy information about availability of computer code

Data collection	Flow cytometry: FACS Diva version 8.0.1 (BD)<br>Mass cytometry: CyTOF vesion 6.7 (Fluidigm)<br>ELISA: Ascent version 2.6 (Thermo Electron)<br>Microscopy: DP controller 3.3.1 (Olympus)<br>Nanostring: nSolver version 4.0 (Nanostring Technologies)<br>RNA-Seq: RTA version 3.3.3<br>Photography of cells: Zen Blue version 2.0.0.0 (Carl Zeiss)
Data analysis	GraphPad Prism version 8.2.1 (GraphPad)<br>Flow cytometry: Flow Jo versions 9.9.6 and 10.7.1 (FlowJo LLC), Cytobank (Cytobak Inc.)<br>Mass cytometry: Cytobank (Cytobank Inc)<br>Nanostring: Partek version 7.0 (Partek), R (R Foundation)<br>RNA-Seq: STAR version 2.5, Subread featureCounts version 1.5.0-p1, RefSeq transcripts (UCSC), DESeq2 version 1.14.1, STAR version 2.5<br>scRNA-Seq: Cell Ranger 3.0.1, Seurat version 3.0 in R version 3.6.2 (http://satijalab.org/seurat/), Monocle 2 (http://cole-trapnell-lab.github.io/monocle-release/docs/)

For manuscripts utilizing custom algorithms or software that are central to the research but not yet described in published literature, software must be made available to editors/reviewers. We strongly encourage code deposition in a community repository (e.g. GitHub). See the Nature Research guidelines for submitting code & software for further information.

Data

Policy information about availability of data

All manuscripts must include a data availability statement. This statement should provide the following information, where applicable:

- Accession codes, unique identifiers, or web links for publicly available datasets
- A list of figures that have associated raw data
- A description of any restrictions on data availability

Data availability has been described in the manuscript. Bulk RNA-Seq data pertain to Figs, 2, 3, Ext. Data Figs. 2, 3 are available from the Gene Expression Omnibus (GEO) (https://www.ncbi.nlm.nih.gov/geo/) (accession number GSE149778). Nanostring data pertain to Fig. 5 and Ext. Data Fig. 5 are available from the GEO (accession number) and provided in the Supplementary Table 2. scRNA-Seq data in Figs. 5, 6, Ext. Data Figs. 5, 6, 7 are available from the GEO (accession number GSE156527). cDC2 cluster DEGs pertain to Fig. 5 is provided in the Supplementary Table 3. Additional raw data are available from the corresponding authors upon request.

Field-specific reporting

Please select the one below that is the best fit for your research. If you are not sure, read the appropriate sections before making your selection.

☒ Life sciences ☐ Behavioural & social sciences ☐ Ecological, evolutionary & environmental sciences

For a reference copy of the document with all sections, see nature.com/documents/nr-reporting-summary-flat.pdf

Life sciences study design

All studies must disclose on these points even when the disclosure is negative.

Sample size	Sample size was determined based on previous studies. 3 to 14 mice per group were sufficient to detect differences between groups with power value 0.8 and significance level 0.05 for most experiments.
Data exclusions	No data were excluded from the analysis
Replication	All experiments were replicated at least 2 independent experiments. The numbers of replication are indicated in figure legends.
Randomization	Age- and sex-matched wild type and knockout or transgenic mice were assigned to different groups in Fig. 1, 4 and Ext. Data Fig. 1, 14.
Blinding	Experiments were not performed in a blind manner. Researchers were aware of genotyping results prior to experiments.

Reporting for specific materials, systems and methods

We require information from authors about some types of materials, experimental systems and methods used in many studies. Here, indicate whether each material, system or method listed is relevant to your study. If you are not sure if a list item applies to your research, read the appropriate section before selecting a response.

Materials & experimental systems

n/a	Involved in the study	
☐	☒	Antibodies
☒	☐	Eukaryotic cell lines
☒	☐	Palaeontology
☐	☒	Animals and other organisms
☒	☐	Human research participants
☒	☐	Clinical data

Methods

n/a	Involved in the study	
☒	☐	ChIP-seq
☐	☒	Flow cytometry
☒	☐	MRI-based neuroimaging

Antibodies

Antibodies used	Following antibodies used for flow cytometry were obtained from BD Biosciences, BioLegend or eBioscience/ThermoFisher Scientific. BUV395-, BV510- or biotin-anti-mouse CD3e (145-2C11), APC- or eFluor450-anti-mouse CD4 (GK1.5), BUV395- or PE-anti-mouse CD11b (M1/70), Alexa Fluor 488-, PerCP-Cy5.5- or PE-Dazzle594-anti-mosue CD11c (N418), APC-Fire750- or BV510-anti-mouse CD14 (Sa14-2), BV510- or biotin-anti-mouse CD19 (605), APC-Cy7-anti-mouse CD44 (IM7), APC-Cy7-anti-mouse CD45.1 (A20), BV510- or BV711-anti-mouse CD45.2 (104), biotin-anti-mouse CD45R-B220 (RA3-6B2), APC-, PE-, PerCP-Cy5.5- or biotin-Cy5.5-CD88 (20/70), APC-, BV510- or PE-anti mouse CD103 (M290), PE-anti-mouse CD135 (A2F10), FITC-anti-mouse CD172a (P84), Alexa Fluor 647-, BV711- or PE-anti-mouse CD200 (OX-90), APC- PE- or PE-Dazzle594-anti-mouse CD301b (URA-1), BUV737- or PE-Dazzle594-anti-mouse F4/80 (T45-2342 or BM8), BV510- or BV711-anti-mouse Ly-6A/E (D7), APC-eFluor780-, BUV395-, BV711-, FITC- or biotin-anti-mouse Ly-6C (AL-21 or HK1.4), biotin-anti-mouse Ly-6G (1A8), eFluor450-anti-mouse MHC class-II I-Ab (AFb.120), APC-, PE-, PerCP-Cy5.5- or biotin-anti-mouse Siglec-F (E50-2440), biotin-anti-mouse TER119 (TER-119),

PE-hamster IgG, APC-, BV510-, BV711- or PE-rat IgG2a, PE-rat IgG2b and FITC-rat IgM. Biotinylated antibodies were follow by streptavidin conjugated with BUV395 or BV510.

Metal-conjugated antibodies used for mass cytometry were obtained from Fluidigm or Lederer Lab (Harvard). Some purified antibodies were purchased from BD Bioscience, BioLegend or eBioscience/ThermoFisher Scientific, and conjugated to metals by Fluidigm. fluorochrome-conjugated antibodies were obtained from BioLegend. Staining with fluorochrome- or biotin-conjugated antibodies was followed by metal-conjugated secondary antibodies. Antibodies used for mass cytometry are listed in Supplementary Table 1.

For enrichment of preDCs from mouse bone marrow, biotin-conjugated anti-mouse CD3e, CD11b, CD19, CD45R-B220, CD49b, Ly-6A/E, Ly-6G and TER119 (BD Biosciences, BioLegend or eBioscience/ThermoFisher Scientific) were used for the negative selection in a magnet activated cell sorter (MACS) (Miltenyi).

For preparation of naive CD4 T cells, biotin-conjugated anti-mouse CD8α (53-6.7), CD8b (53-5.8), CD11b (M1/70), CD11c (HL3), CD16/32 (2.4G2), CD19 (605), CD25 (PC61), CD44 (IM7), CD45R-B220 (RA3-6B2), CD49b (DX5), MHC class-II I-Ab (AFb.120), and Ly-6C/G (RB6-8C5) antibodies were obtained from BD Biosciences, BioLegend or eBioscience/ThermoFisher Scientific.

For T cell activation in vitro, Purified anti-mouse CD3e (145-2C11) and CD28 (D665 or 37.51) were obtained from BD Bioscience or eBioscience/ThermoFisher Scientific.

Validation

All antibodies are commercially available, and validated by manufacturer and previous studies. Fluorescence-, biotin- and metal-conjugated antibodies were validated by vendors.

Animals and other organisms

Policy information about studies involving animals; ARRIVE guidelines recommended for reporting animal research

Laboratory animals

C57BL/6J, Batf3–/– (B6.129S(C)-Batf3tm1Kmm/J), Ccr2–/– (B6.129S4-Ccr2tm1Ifc/J), Cd11c-Cre (B6.Cg-Tg(Itgax-cre)1-1Reiz/J), CD45.1 (B6.SJL-Ptprca Pepcb/BoyJ), DTA-fx (B6.129P2-Gt(ROSA)26Sortm1(DTA)Lky/J), Lta–/– (B6.129S2-Ltatm1Dch/J), OT-II TCR transgenic (B6.Cg(TcraTcrb)425Cbn/J) and zDC-DTR (B6(Cg)-Zbtb46tm1(HBEGF)Mnz/J) mice were purchased from Jackson Laboratories. Flt3L–/– (C57BL/6-Flt3Ltm1/mx) and Cx3cr1–/– (B6.129-Cx3cr1tm1Zm) mice were purchased from Taconic Biosciences (Germantown, NY, USA). OVA-specific Il17a fate-mapping mice (B6.Cg-Il17atm1.1(EYFP/cre)Ehs Gt(ROSA)26Sortm9 (CAG–tdTomato)Hze Tg(TcraTcrb)425Cbn) were generated in our lab. CD45.1-OT-II mice were generated by crossing CD45.1 and OT-II TCR transgenic mice. Ccr2–/– Cx3cr1–/– DKO mice were generated by crossing Ccr2–/– and Cx3cr1–/– mice. ΔDC mice were generated by crossing Cd11c-Cre and DTA-fx mice. Batf3–/– ΔDC mice were generated by crossing Batf3–/– Cd11c-Cre and Batf3–/– DTA-fx mice. Mice were bred and housed in specific pathogen-free conditions at the NIEHS, and used between 6 and 12 weeks of age. Age- and sex-matched mice were used for experiments.

Wild animals

Wild animals were not used in this study.

Field-collected samples

not applicable.

Ethics oversight

All mouse studies were performed in accordance with guidelines provided by the Institutional Animal Care and Use Committees in the NIEHS.

Note that full information on the approval of the study protocol must also be provided in the manuscript.

Flow Cytometry

Plots

Confirm that:

- [x] The axis labels state the marker and fluorochrome used (e.g. CD4-FITC).
- [x] The axis scales are clearly visible. Include numbers along axes only for bottom left plot of group (a 'group' is an analysis of identical markers).
- [x] All plots are contour plots with outliers or pseudocolor plots.
- [x] A numerical value for number of cells or percentage (with statistics) is provided.

Methodology

Sample preparation

For DC preparation, minced tissues were digested for 60 min with Liberase, Collagenase XI, Hyaluronidase and DNase. To enrich DCs, low density cells in the lung cells were collected by gradient centrifugation using 16 % Nycodenz (Accurate Chemical).

For T cell preparations, lung or mLNs were digested for 30 min, and single cell suspension generated. To enrich T cells, mononuclear cells were enriched by gradient centrifugation using Histopaque 1083 (Millipore Sigma).

To isolate preDCs, bone marrow was collected from femurs, tibia, humeri and sternum bones, and red blood cells lysed with ACK buffer containing 0.15 M ammonium chloride and 1 mM potassium bicarbonate. Cells were passed through cell strainers, and mononuclear cells were enriched by gradient centrifugation using Histopaque 1083. PreDCs were enriched using an automated magnet-activated cell sorter (AutoMACS, Miltenyi) by negative selection using antibodies against CD3, CD11b, CD19, CD45R-

	B220, CD49b, Ly-6A/E, Ly-6G and TER119.<br><br>Cells were diluted to one million cells/100 µL and incubated with a non-specific binding blocking reagent cocktail of anti-mouse CD16/CD32 (2.4G2), normal mouse and rat serum (Jackson ImmunoResearch). Cell surface antigens were stained with fluorochrome- or biotin-conjugated antibodies. Staining with biotinylated antibodies was followed by fluorochrome-conjugated streptavidin.
Instrument	FACS LSR Fortessa (BD) with 5 lasers was used for analysis. FACS ARIA-II (BD) with 5 lasers was used for cell sorting.
Software	Data collection: FACS Diva version 8.0.1 (BD)<br>Data analysis: Flow Jo versions 9.9.6 and 10.7.1 (FlowJo LLC) and Cytobank (Cytobak Inc)
Cell population abundance	The purify of post-sort fractions (>90%) was verified by re-analysis in flow cytometry.
Gating strategy	Gating strategies for analysis of lung cDC subsets, monocytes and macrophages are depicted in Supplementary Fig. 1d and 3a,b. cDCs were Live/Dead– CD45+ CD11c+ I-A+ CD88– Siglec-F– F4/80– singlets. Monocytes were Live/Dead– CD45+ F4/80+ CD88– Siglec-F– singlets. Alveolar macrophages were Live/Dead– CD45+ CD11c+ Siglec-F+ singlets. Interstitial macrophages were Live/Dead– CD45+ CD88+ F4/80+ Siglec-F– singlets.<br><br>Gating strategies for preDCs are depicted are Supplementary Fig. 5a,b. FSH-A and FSC-H were used to identify singlets. FSC-A and SSC were used to exclude debris. Lineage (CD3e, CD19, B220, Ly-6G and TER119)-negative CD11b-nagative cells were selected. CD11c+ I-A– cells were selected, and CD135+ and CD172a intermediate cells were designated as preDCs.<br><br>Gating strategies for antigen uptake by cDCs are depicted in Supplementary Fig. 6a,b. cDC2 were Live/Dead– CD45+ CD11b+ CD11c+ I-A+ CD88– Siglec-F– F4/80– singlets.<br><br>Gating strategy for ex vivo culture of cDCs and monocytes is depicted in Supplementary Fig. 9a. cDC2 were Live/Dead– CD45+ CD11b+ CD11c+ I-A+ CD88– Siglec-F– F4/80– singlets. Monocytes were Live/Dead– CD45+ F4/80+ CD11b+ Ly-6C+ CD88– Siglec-F– singlets.<br><br>Gating strategies for cDC2 subpopulation analysis are depicted in Supplementary Fig. 11a,b. cDC2 were CD11b+ CD11c+ I-A+ CD14– CD88– Siglec-F– F4/80– singlets.<br><br>Gating strategies for cDC2 subpopulation analyses in adoptive transfer experiments are depicted in Supplementary Fig. 13a,b. In cell sorting, cDC2 were CD11b+ CD11c+ I-A+ CD14– CD88– Siglec-F– F4/80– singlets. Donor cDC2-derived cDC2 were CD45.2+ CD11b+ CD11c+ I-A+ CD88– Siglec-F– F4/80– singlets.<br><br>Gating strategy for cDCs migration analysis is depicted in Supplementary Fig. 16a. cDC2 were CD3e– CD19– CD11b+ CD11c+ I-A+ CD88– Siglec-F– F4/80– singlets.<br><br>Gating strategies for CD4 T cell analysis are depicted in Supplementary Fig. 16a,b. FSH-A and FSC-H were used to identify singlets. FSC-A and SSC were used to exclude debris. After gating on Live/Dead– CD4+ cells, CD3e+ CD45.2+ cells were designated as CD4 T cells derived from OT-II transgenic donor mice (CD45.2).

☒ Tick this box to confirm that a figure exemplifying the gating strategy is provided in the Supplementary Information.
